# Experiments Investigating the Competitive Growth Advantage of Two Different Genotypes of Human Metapneumovirus: Implications for the Alternation of Genotype Prevalence

**DOI:** 10.1038/s41598-020-59150-9

**Published:** 2020-02-18

**Authors:** Zhen Zhou, Pan Zhang, Yuxia Cui, Yongbo Zhang, Xian Qin, Rongpei Li, Ping Liu, Ying Dou, Lijia Wang, Yao Zhao

**Affiliations:** 1 Department of Pediatric Research Institute; Ministry of Education Key Laboratory of Child Development and Disorders; National Clinical Research Center for Child Health and Disorders; China International Science and Technology Cooperation base of Child development and Critical Disorders, Children’s Hospital of Chongqing Medical University, Chongqing, P.R. China; 2 Chongqing Key Laboratory of Child Infection and Immunity, Chongqing, 400014 China; 3 Department of Pediatrics, Guizhou Provincial People’s Hospital, Guizhou, 550002 China

**Keywords:** Genotype, Infection

## Abstract

Human metapneumovirus (hMPV) is an important pathogen that causes upper and lower respiratory tract infections in children worldwide. hMPV has two major genotypes, hMPV-A and hMPV-B. Epidemiological studies have shown that the two hMPV genotypes alternate in predominance worldwide in recent years. Co-circulation of the two genotypes of hMPV was usually observed and there is no study about the interaction between them, such as competitive replication, which maybe the possible mechanisms for alternating prevalence of subtypes. Our present study have used two different genotypes of hMPV (genotype A: NL/1/00; B: NL/1/99) in different proportions in animal model (BALB/c mice) and cell model (Vero-E6) separately. The result showed that the competitive growth does exist in BALB/c mice, genotype B had a strong competitive advantage. However, genotype B did not cause more severe disease than non-predominant (genotype A) or mixed strains in the study, which were evaluated by the body weight, airway hyperresponsiveness and lung pathology of mouse. In cell model, competitive growth and the two genotypes alternately prevalence were observed. In summary, we confirmed that there was a competitive replication between hMPV genotype A and B, and no difference in disease severity caused by the two subtypes. This study shows a new insight to understand the alternation of hMPV genotype prevalence through genotype competition and provide experimental evidence for disease control and vaccine design.

## Introduction

Human metapneumovirus (hMPV), which was first isolated from children with respiratory tract infections (RTIs) in the Netherlands in 2001 and belongs to the Paramyxoviridae family and Pneumovirus subfamily^1^, is a leading cause of respiratory infections in children worldwide^2,3^. hMPV has been recognized as a causative agent of respiratory infections, including upper respiratory infections, severe bronchiolitis and pneumonia, in all age groups, with more severe disease occurring in infants, elderly people, and immunocompromised hosts^4–7^. The epidemic season of hMPV is reported to occur from winter to early spring^8^. There are no effective vaccines or drugs to combat hMPV infection.

hMPV has been speculated to have similar characteristics as human respiratory syncytial virus (RSV), and this has been gradually confirmed by research over the last 18 years^8–10^. The seasonality, clinical manifestations, and age distribution of hMPV infection resemble those of RSV infection, and the alternating pattern of the two genotypes of hMPV shows similar properties^11^. hMPV is a negative sense, single-stranded RNA virus with a genome that is approximately 13 kb in length that encodes eight genes and nine proteins, including the following genes and proteins, respectively: N, nucleocapsid; P, phosphoprotein; M, matrix; F, fusion; SH, small hydrophobic; G, glycoprotein; and L, polymerase and M2-1 and M2-2. Based on the genetic variability and phylogenetic analysis of the G gene and the F gene, hMPV is classified as one of two major genotypes, hMPV-A and hMPV-B, which are subdivided into four subgroups (A1, A2, B1 and B2)^12,13^.

Epidemiological surveys conducted in many countries, such as China, the USA, France, Australia, Italy, Korea, Brazil, and India, have indicated that strains from both major hMPV groups co-circulate within the same community and that the predominant subgroup shifts progressively from one group to another. Many epidemiological studies have found that A/B genotyping indicated a change in the predominant viral genotype over one to three years^2,14–20^. Theo P *et al*. reported that hMPV subtype A was predominant in 2001–2003, but this shifted to subtype B in 2004 in Queensland, Australia^21^; Wuhua Kong found that genotype A prevailed in the epidemic seasons in 2008–2009 and 2012–2013, while genotype B prevailed in 2009 in Wuhan, China^22^; an article published in Korea in 2016 about an epidemic of hMPV showed that genotype A2a was predominant in 2007 and 2010, genotype B1 was predominant in 2012, and B2 was predominant in 2008 and 2009 and further predicted that genotype A2a would be the predominant hMPV type in 2014 or 2015 in Korea^23^.

Despite the common worldwide of hMPV alternating epidemics, the underlying mechanisms are not well understood. Many researchers have suggested that virus alternating epidemic may be related to the environmental-climate change, host immunity and susceptibility, and the bottleneck effect of the virus transmission process^24–26^. The above reasons are “external factors” of this alternating epidemic, “internal factors” include virus escape, virus evolution, and interactions between virus subtypes. Co-circulation of the two genotypes of hMPV was usually observed and the interactions between them such as competitive growth might play a role in the formation of alternation^22,27,28^. However, there is no study about it. Most studies of the alternation of the A/B strains during epidemics have been focused on clinical investigations of hMPV strains isolated from symptomatic patients that involved genotyping and statistical analysis, and there was no basic experimental evidence^29–31^; We set our study in animal model (BALB/c mice) and cell model (Vero-E6) separately with two different genotypes of hMPV (genotype A: NL/1/00; B: NL/1/99) in different proportions.

This study confirmed that there was a competitive replication between genotype A and genotype B. In animal experiment, subtype B became the predominant strain, but the clinical symptoms caused by type B were not more severe than non-predominant or mixed strain. In cell experiment, type A and B alternately predominated at the first four generations, and A became the predominant strain eventually. This research could offer a new opinion to study alternation of genotype prevalence. Our findings provide important information for treatment research, vaccine development, prevention strategies, and the monitoring of outbreaks of hMPV infection.

## Results

### *In vivo* competitive growth experiment

**Figure 1:**
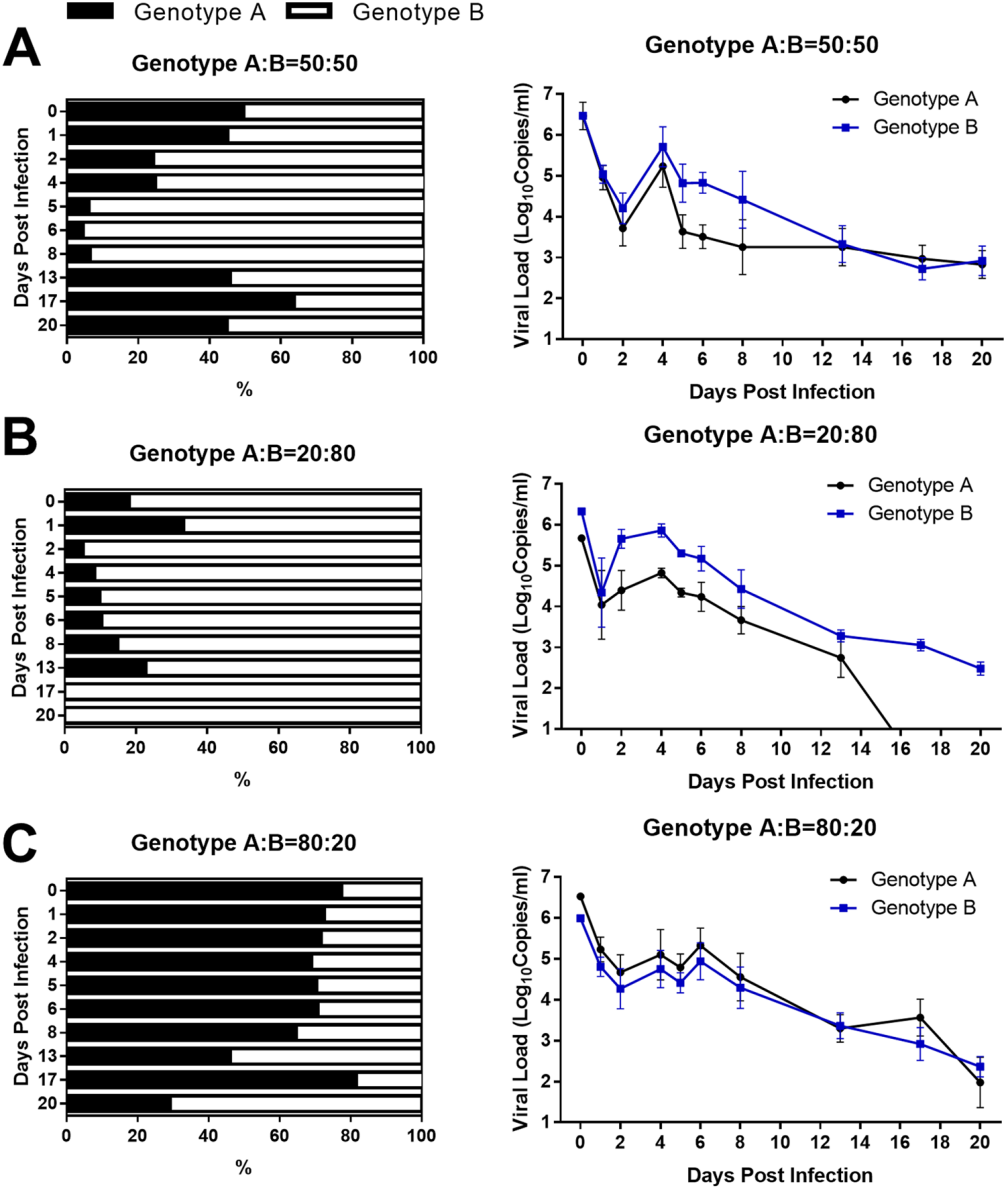
*In vivo competitive growth experiment*. The BALB/c mouse model was used for the hMPV infection model, and each mouse was infected with 50 μl of virus at 1.0 × 10^9^ copies/μl through the nose. There were 3 groups with different genotype A:B proportions: 50:50, 20:80, and 80:20. (**A**) The input ratio of genotype A:B = 50:50. (**B**) The input ratio of genotype A:B = 20:80. (**C**) The input ratio of genotype A:B = 80:20. All the figures on the left show the ratio change in each passage post infection (Black represents genotype A, and Blank represents genotype B) and the figures on the right show the viral load of the two genotypes (Solid circles represent genotype A, and solid squares represent genotype B).

To determine whether competitive replication occurred *in vivo*, we divided the mice into six groups. Three groups were given nasal drops with different proportions of mixed strains (genotype A:B) as 50:50, 20:80 and 80:20; Two groups dropped with genotype A and genotype B; one group dropped with DMEM (Virus preservation solution) as control group. If the copy numbers were too low, the detection accuracy could not be guaranteed. When the virus copy numbers in the lung were less than 10^3^, the competitive experiment was terminated. A similar alternation in terms of the predomination of genotypes A and B could be seen in the mouse model. When A:B = 50:50 (Fig. 1A), the proportion of genotype A decreased from day 1 to 8, but after day 8, it increased from 4.54% (day 6) to 63.74% (day 17); by day 20, it had decreased to 44.90%, and genotype B was predominant.

After the adjustment of the input ratio of the A:B genotypes to 20:80 (Fig. 1B), the proportion of A was increased on day 1 and decreased from day 2 to day 8; however, during this process, the proportion of A increased slowly and was higher than 20% on day 13. Regarding the group with an input ratio of 80:20 (Fig. 1C), the two genotypes alternated in terms of their proportions. The proportion of genotype B increased slowly and was higher than 50% on day 13, but decreased on day 17, then increased to 72% on day 20. In a word, hMPV can only survive for 3 weeks in mice. Regardless of the initial proportion of subtype B, after competing with subtype A, type B would dominate finally and show an absolute growth advantage.

### Weight change in mice with hMPV infection

**Figure 2:**
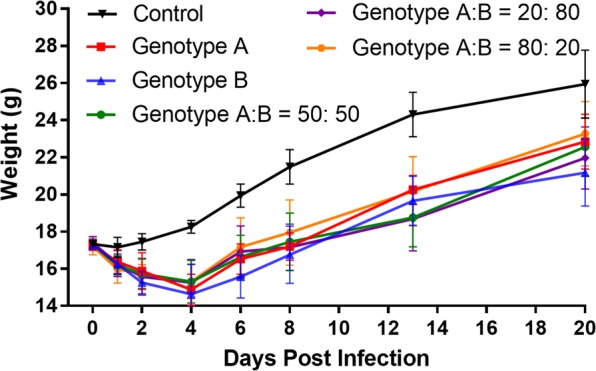
Weight change in mice with hMPV infection. Control group: Black solid inverted triangles; Genotype A alone: Red solid squares; Genotype B alone: Blue solid triangles; Genotype A:B = 50:50: Green solid circles; Genotype A:B = 20:80: Purple solid diamonds; Genotype A:B = 80:20: Orange solid squares. P > 0.05 for the comparison among the five infected groups. P < 0.05 for the comparisons of the control group with the infected groups.

BALB/c mice were infected with 50 μl of hMPV virus (1.0 × 10^9^ copies/ml) and were observed daily to measure their weight loss. Starting 1 dpi (days post infection, dpi), the mice began to have ruffled hair that persisted until 6 dpi. Breathing problems appeared 1 to 6 dpi, as well as a slight decrease in physical activity and a tendency to huddle. Significant weight loss appeared 4 dpi (P < 0.05). In the control group, neither weight loss nor respiratory symptoms were observed (Fig. 2). There were no statistically significant differences after 20 days of infection between the groups (in the genotype A alone, genotype B alone and 3 mixed genotype groups) (P > 0.05). Differences only appeared in the infection and control groups on the 2nd day after infection.

### Pulmonary virus titres

**Figure 3:**
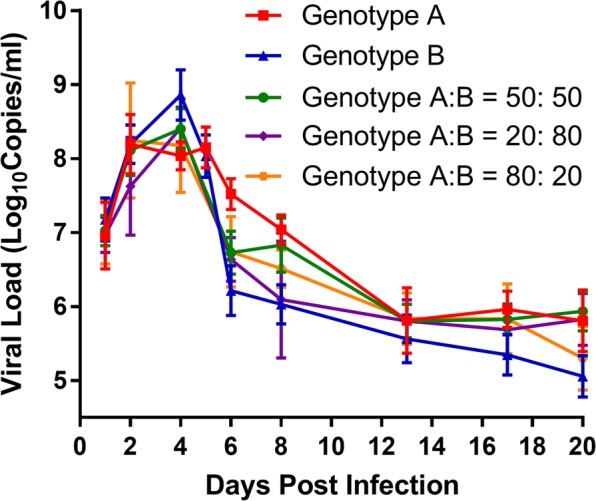
Pulmonary virus titre change in BALB/c mice. The viral titre in the lungs of hMPV-infected BALB/c mice increased gradually and peaked at approximately 3.0 × 10^8^ copies/ml 4 dpi and was still detectable at approximately 5.0 × 10^5^ copies/ml 20 dpi. There were no differences among the three infected groups (P > 0.05). Genotype A alone: Red solid squares; Genotype B alone: Blue solid triangles; Genotype A:B = 50:50: Green solid circles; Genotype A:B = 20:80: Purple solid diamonds; Genotype A:B = 80:20: Orange solid squares.

The viral titre (total viral load of genotype A and genotype B) in the lungs of hMPV-infected BALB/c mice increased gradually and peaked at 4 dpi at approximately 3.0 × 10^8^ copies/ml and was still detectable at 20 dpi (approximately 5.0 × 10^5^ copies/ml). There were no differences among the five infected groups (P > 0.05) (Fig. 3).

### Airway responsiveness

An increase in breathing problems appeared at 1 to 6 dpi in BALB/c mice when compared to the DMEM control group, and higher respiratory rates and scratching of noses was observed. Four days after infection, the airway responsiveness was tested.

**Figure 4:**
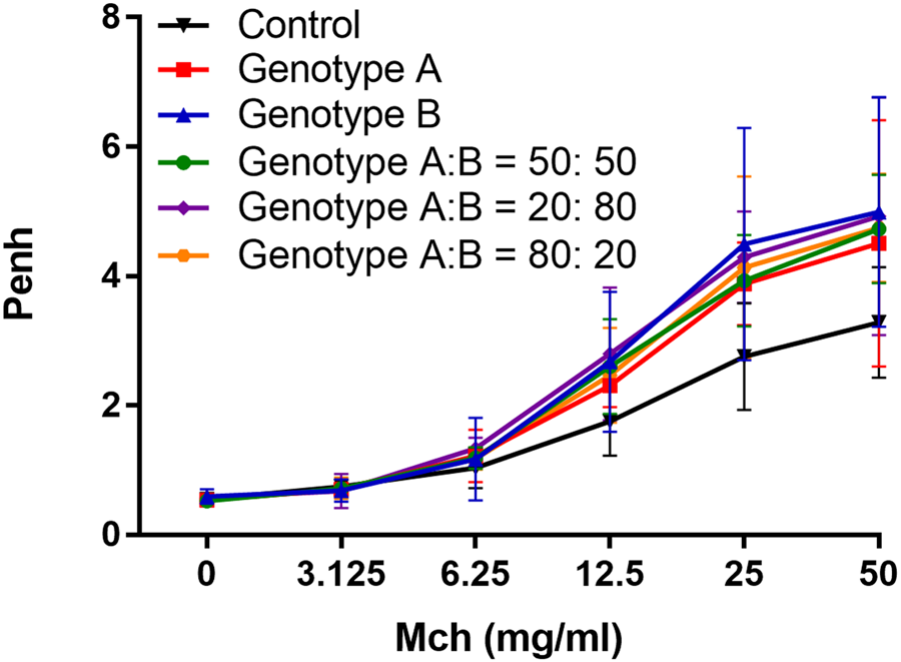
Airway responsiveness of BALB/c mice. Lower dosages of methacholine (3.125 and 6.25 mg/ml) produced no differences (P > 0.05) between the control group and the infected groups, but higher doses of methacholine (12.5, 25, and 50 mg/ml) produced differences (P < 0.05). There were no differences among the five infected groups (P > 0.05). Control group: Black solid inverted triangles; Genotype A alone: Red solid squares; Genotype B alone: Blue solid triangles; Genotype A:B = 50:50: Green solid circles; Genotype A:B = 20:80: Purple solid diamonds; Genotype A:B = 80:20: Orange solid squares.

The results (Fig. 4) showed that there were no differences between the control group and any of the infection groups when dosages of methacholine of 3.125 and 6.25 mg/ml were administered (P > 0.05). However, when the concentration of methacholine was higher than 12.5 mg/ml, there was a significant difference between the infection groups and the control group (P < 0.05). At concentrations of 25 and 50 mg/ml, there was significantly higher airway responsiveness in all infection groups than in the control group (P < 0.05). Nevertheless, there were no differences among the five infected groups (P > 0.05).

### Histopathological changes in the lungs

**Figure 5:**
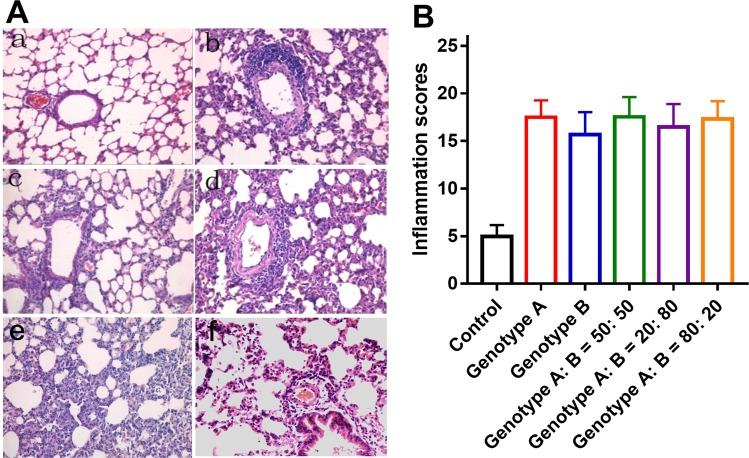
Histopathological changes in the lungs of BALB/c mice. The lung histopathology in each group; six mice were sacrificed at 5 dpi, and their lungs were removed and fixed with 10% formalin. Thin sections of paraffin-embedded lung tissues were cut and stained with haematoxylin and eosin. (**A**) Representative sections (magnification 200x) are shown. Compared with non-infected mice, all groups of infected mice showed swelling of bronchiolar epithelial cells, alveolar dilation and extensive infiltration of lymphocytes and macrophages surrounding the bronchioles and higher mean scores. a-f represent the control group, genotype A alone, genotype B alone, genotype A:B = 50:50; genotype A:B = 20:80 and genotype A:B = 80:20. (**B**) Inflammation scores for lung histopathology in BALB/c mice. The inflammatory response was significantly more dramatic compared to that in the DMEM control group (P < 0.01). In addition, there were no differences among the five infected groups (P > 0.05).

Pulmonary inflammation was assessed using a scoring scale system developed by Cimolai *et al*.^28^. The highest viral load was observed on the fourth day, and the mice were sacrificed on the fifth day to assess the pathological changes. On the 5th day, a few infiltrating cells were found around bronchioles or vessels in non-infected mice, and infected mice showed swelling of the bronchiolar epithelial cells, alveolar dilation and extensive infiltration of lymphocytes and macrophages in both bronchioles and pulmonary blood vessels, indicating a high mean score (Fig. 5A). The inflammatory responses in the infection groups were significantly more dramatic compared to those in the DMEM control group (P < 0.05). However, there were no differences among the five infected groups (P > 0.05) (Fig. 5B).

### In vitro competitive growth experiment

Vero-E6 cell lines were used for the hMPV competitive experiment *in vitro*, and the same multiplicity of infection (MOI = 50) was used for all infection groups. There were 5 mixed groups with genotype A:B proportions of 50:50, 20:80, 80:20, 10:100 and 100:10. The cells were passaged every 4 days. The competitive experiment was terminated when one of the genotypes could not be detected for at least three passages.

**Figure 6:**
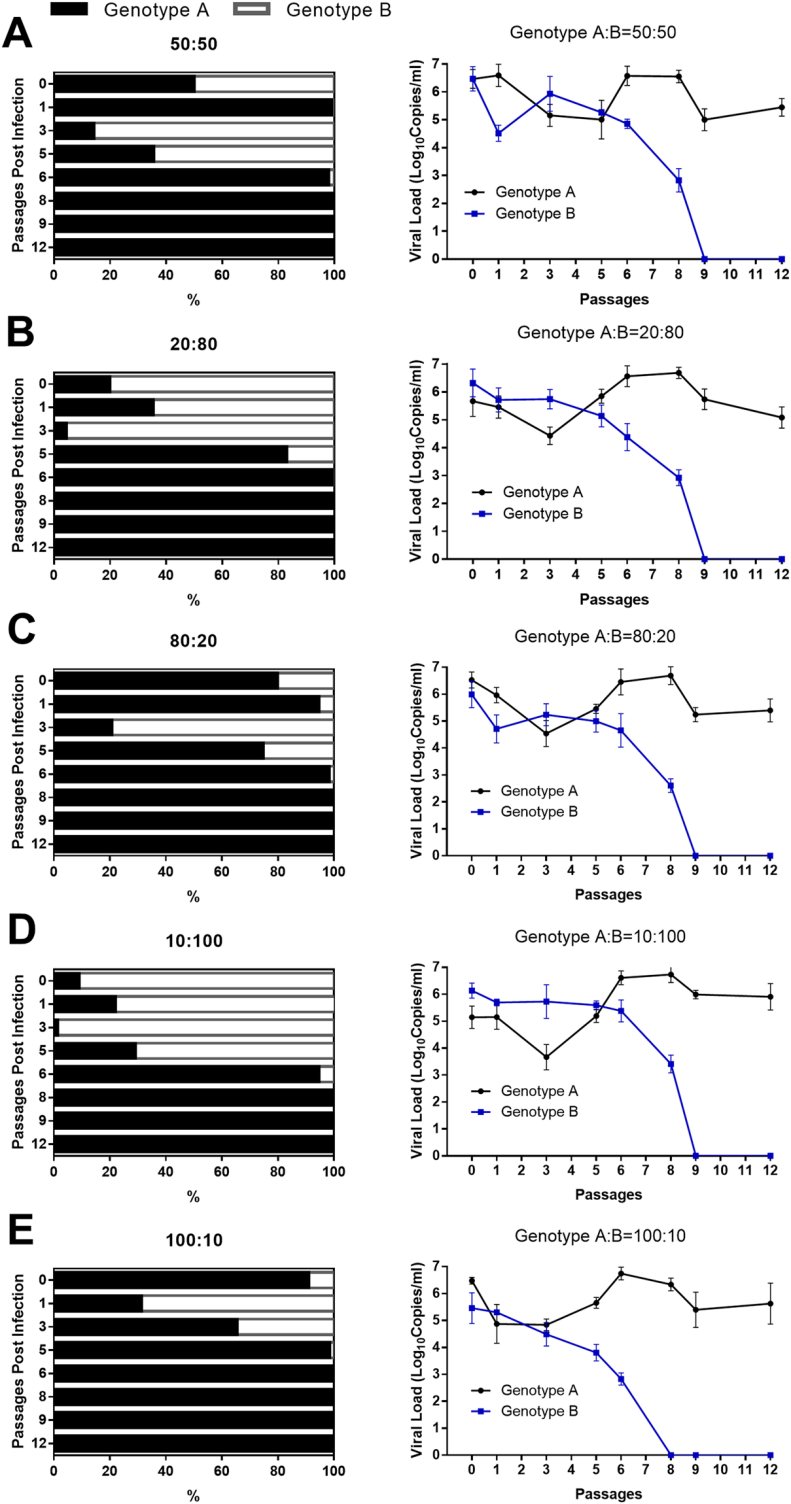
*In vitro* competitive growth experiment. Vero-E6 cell lines were used for the hMPV competitive experiment. The MOI of infection was 50. There were five mixed genotype groups: 50:50, 20:80, 80:20, 10:100 and 100:10 (Genotype A:B). Cells were passaged every 4 days. (**A**) The input ratio of genotype A:B = 50:50. (**B**) The input ratio of genotype A:B = 20:80. (**C**) The input ratio of genotype A:B = 80:20. (**D**) The input ratio of genotype A:B = 10:100. (**E**) The input ratio of genotype A:B = 100:10. All the figures on the left show the ratio change in each passage post infection (Black represents genotype **A**, and Blank represents genotype **B**) and the figures on the right show the viral load of the two genotypes (Solid circles represent genotype A, and solid squares represent genotype B).

First, the input ratio of the two genotypes was adjusted to 50:50. Genotype A predominated (99.08%) during the 1st passage, which was followed by a decrease in the 3rd passage, while genotype B was predominant (85.68%) in 3rd passage (Fig. 6A). Interestingly, after the 3rd passage, the proportion of genotype A gradually increased from 14.32% (passage 3, P3) to 35.67% (P5) to 98.07% (P6) and then finally to 99.98% (P8) until B could not be detected. Competition replication of this group was from type A to B and genotype A become the predominant strain in the end.

When the hMPV input ratio was adjusted to A:B = 80:20 and 100:10, subtype A account for a larger proportion at first. In Fig. 6C, during P1, the proportion of genotype A increased from 80% to 94.71%. However, at P3, the proportion of B increased, and B predominated (79.19%). Then, the proportion of B decreased from 25.22% (P5) to 1.57% (P6) until B was not detected (P8). A 10-fold difference in the input ratio was utilized to determine whether the competition between the two genotypes could still be observed. When the proportion of genotype A was ten-fold that of B (Fig. 6E), competition of the two subtypes were also observed, genoype A decreased and then increased to be predominant strain.

The proportion of subtype infection (genotype A:B as 20:80, 10:100) was controlled to make B subtype dominant in the firstly passage. In genotype A:B = 20:80 group (Fig. 6B). During the 1st passage, the proportion of genotype A rose to 34.49% but then decreased to 4.56% (P3). At the same time, the number of viral copies was maintained at approximately 5 × 10^4^. Then, the proportion of genotype A increased to 83.03% at P5 and then further increased to 99.32% (P6) and 99.98% (P8). After P9, genotype B could no longer be detected at any copy number. In genotype A:B = 10:100 group (Fig. 6D), even when the proportion of genotype A was only one-tenth of that of B, the result was the same: the proportion of genotype A increased, decreased and increased again until we could not detect genotype B (P9). Even in the two groups with a high proportion of subtypes B, there will be a competition and alternation phenomenon first and finally genotype A will reverse to become the dominant strain.

## Discussion

hMPV is an important cause of acute respiratory illness in young children. Since it was discovered in the Netherlands in 2001, hMPV has been identified in 3–10% of hospitalized young children and has been found all over the world^1,10^. A 10-year follow-up study on hMPV found that the annual prevalence rate ranged from 5.5–12% in Belgium between 2006 and 2016 based on the assessment of 16826 respiratory samples^32^. hMPV was most frequently discovered in late winter and spring. Two main hMPV types have been recognized (A and B), and each type has 2 subtypes (A1, A2; B1, B2)^33–35^. Research on the regular epidemiological pattern of hMPV showed that there is usually a predominant genotype of the circulating hMPV strain.

Much evidence has been gathered about the alternation of genotypes A and B. Agapov. E proposed for the first time between 2002 to 2004, the predominant circulating genotype shifted from A to B in the USA^2^. In eastern India, both group A and B viruses co-circulated in 2006–2007, but later B disappeared and genotype A became the only epidemic subtype^20^. In Korea, the predominant genotype shifted from A2a to B2 between 2007 and 2010 and then shifted back to A2b^18^. A similar alternation was also observed in Malaysia from 2010 to 2012, during which the genotype shifted from A2b to B1 and then to A2a^36^. The predominant genotype was B1 in 2004–2005, which was followed by a shift to the A2 genotype in 2006 in southeastern Brazil^19^. We also have found some reports regarding the phenomenon of subtype A/B alternation in China. Zhang C *et al*. observed a shift from the A2b, A1 and B1 genotypes (2008 to 2009) to the A2b genotype (2009–2011) in Chongqing^14^. These observations were consistent with those made in Beijing, which indicated that A2, B1 and B2 co-circulated, and A2 was the most prevalent genotype from 2008 to 2010^37^. The most prevalent genotype was A in 2008–2009, which shifted to B in 2010–2011 and then shifted back to A in 2012–2013 in Wuhan^22^.

We analysed the global trends in terms of hMPV subtypes, and the statistical results were consistent with those of a previous study that reported an alternating trend in the global hMPV subtypes over ten years^38^. The statistical results for the two subtypes indicated that subtypes A and B of hMPV alternate in terms of prevalence, and each epidemic can last for 1–3 years. This result was also compared with those of previous studies^2,14–20,29,39,40^. Many researchers have studied the alternation of genotype prevalence of hMPV and the most discussed reason for this phenomenon is environmental changes and human immunity^24,41^. Viral evolution and escape are also thought to play a role in this phenomenon^42,43^. In summary, studies of the epidemiological distribution, alternation of the genotype predominance and genetic diversity of hMPV have been conducted all over the world, but these studies have mainly involved patients with clinical manifestations of respiratory infection, and no basic research has been conducted to explain the occurrence of this phenomenon^32,44^. Studies have shown that such alternation may be related to virus-to-host immune escape, and persistent infection or a high viral load is easily detected, which leads to the neglect of low-load subtypes. The differences in the genetic structure of hMPV and immunization against viruses caused a seasonal shift in the predominant genotype and led to the maintenance of infection rates^2,19,21,35,45,46^. If we can determine how hMPV genotypes interact, this may be helpful for epidemic detection, treatment and prevention. We try to focus on the virus itself firstly and explore whether the interaction mechanism between A and B subtypes will affect this alternation.

In the present study, we sought to determine whether the competition exist between A and B subtypes *in vivo* and *in vitro*, and a competitive replication system was used. First, in the mouse model (Fig. 1), the trend of type A was down-up-down, and genotype B was predominant in the end. Except for when A:B = 80:20 during which the starting amount of genotype B was too small, the proportion of B increased slowly in the first 13d and decreased at 17d, and then increased at 20d. There may seem to have a phenomenon that no matter how virus input ratio was used, competition existed between the two subtypes and genotype B predominated in the animal models eventually. We speculated that the competitive advantage of subtype B may be related to its strong virulence^47,48^, so we designed experiments to infect mice with A and B subtype or mixed subtypes to detect the severity of disease. However, based on our *in vivo* animal experiment results, the severity of illness was not related to infection with genotypes A and B in terms of weight changes (Fig. 2), viral load (Fig. 3) airway hyperresponsiveness (Fig. 4) or histopathological changes in the lungs (Fig. 5). This was shown not only for genotype A and B infection alone but also for 50:50, 20:80 and 80:20 mixed genotype infections. Although the results surprised us, previous clinical studies have shown that there is no difference in clinical manifestations between the two subtypes. From 2002 to 2004 in the USA, Apapov E *et al*.^2^ first found that hMPV caused a viral genotype shift, but did not observe a difference in the severity of illness caused by various hMPV isolates. In Italy, from 2003 to 2004, Bosis S *et al*.^49^ found that a high hMPV viral load was correlated with disease presentation, whereas the overall clinical and socioeconomic burden caused by infection with the two hMPV genotypes was similar. From 2006 to 2008 in southern Brazil, Debur MC *et al*. found no correlation between genotype and disease severity in inpatients and outpatients^50^. In the same period (2006–2008) in China, no association was found between hMPV genotypes and disease severity^51^. In summary, we found that hMPV subtypes compete *in vivo*, but virulence is not the reason for its competitive advantages. The specific reasons need to be further explored, but we verified there is a competition between type A and B in animal at the first time, and this competition may play an alternating role in nature.

Then we performed a subtype competitive replication experiment in cells, and wanted to verify this phenomenon *in vitro*. In the cell model (Fig. 6), different input ratios were used to infect Vero-E6 cells, and the viral titer of the two genotypes were detected. Genotype shifts were observed at input ratios of 50:50, 20:80 80:20, 10:100 and 100:10. The proportion of genotype A rose, fell and finally rose again until genotype B could not be detected. Even when A:B = 10:100 and the initial proportion of A was only 1/10 of that of B, it was also possible to gradually obtain a growth advantage and finally for A to become the predominant genotype. In the A:B = 100:10 group, due to the absolute initial proportion of genotype A being very large, the proportion of genotype A did not increase and decline but first decreased and then increased. Based on the above, in spite of the A and B genotypes having different initial proportions, the genotype ratio trends were the same; that is, the proportion of A first increased and then decreased and finally predominated. At the same time, the proportion of genotype B decreased, which was followed by an increase, until finally B disappeared at the detection end point. In summary, we observed the competition of the two hMPV genotypes, type A and B were alternately predominant in cell in the first four generations, and subtype A became predominant strain in the end.

There has been a contradictory phenomenon *in vivo* and *in vitro*; genotype alternation predominated was observed first and then subtype A became the predominant strain in the cell model, but genotype B predominated in animal experiment. We speculate that the reason for this disparity may be that the cell infected with virus as a simple model, and the final result is only related to the different mixing ratios of the hMPV two subtypes. In animal experiments, even if we control the external environment and diet of each group to be the same, the final result is related to the virus subtype addition ratio and the host immunity. Obviously, in animal experiments, subtype B shows a stronger adaptability. This is inconsistent with the conclusion that subtype A is the dominant strain in human^52,53^. It may be that the hMPV dominant strain is different in different populations, or that our experiment time is too short^54,55^. In a word, our experiments showed that the competition exist between the two subtypes and this competition could affect the alternation of genotype prevalence.

Regrettably, there were still several limitations in our experiments. First, we verified the phenomenon using a single model. We used only the Vero-E6 *in vitro* system and BALB/c mice for the *in vivo* experiments, and additional cell lines and animal models needed to be studied. Second, we monitored the animal model for only 20 days, because in previous studies, we found that the hMPV can only survive in mice for 2–3 weeks. If we find a way to extend the survival time of the hMPV in mice, the results may be more consistent with the *in vitro* results. Third, in animal experiments, host immunity can interfere with the impact of hMPV subtype competition on hMPV alternating epidemics. We tried to use the immunosuppressant cyclophosphamide to suppress the immunity of mice, but the use of the inhibitor caused a large number of deaths in mice. We are still looking for suitable inhibitors to suppress the immunity of mice to verify the effect of hMPV subtype competition on alternating epidemics simply. Fourth, the statistical analysis of hMPV global epidemiology may be incomplete, and some reports did not subdivide the subtypes, which may have led to insufficient data for the statistical analysis. Therefore, the actual relationships between hMPV epidemiology, disease severity and genotype alternation need to be further explored.

In summary, we found and verified that the competition between A and B subtypes exists and this competition may relate to the alternation of hMPV genotype prevalence through cell and animal experiments. Regardless of the proportions of genotypes A and B, there will be a subtype become predominant strain in the end. Subtype A predominant *in vitro* and subtype B *in vivo* experiment, and the genotype alternation observed *in vitro*. There were no statistically significant differences in the severity of disease caused by the two hMPV genotypes, and the severity of disease caused by mixed infection was not worse than that caused by single genotype infection. Our results may improve the understanding of the alternation of the prevalence of hMPV genotypes; perhaps this is a strategy involving genetic structure changes that is used by the virus to seasonally maintain infection rates. This study of hMPV affords us the possibility of another explanation of the hMPV alternation epidemic besides the cause of immunity, environment and climate, and it also provides experimental evidence useful for disease control and vaccine development.

## Materials and Methods

### Cell culture

Vero-E6 (ATCC CRL-1586) cells were maintained in Dulbecco’s modified Eagle medium (DMEM) supplemented with 10% foetal bovine serum (Invitrogen, USA), 2 mM L-glutamine, 100 U/ml penicillin and 100 μg/ml streptomycin in an incubator with 5% CO_2_ at 37 °C.

### Viral culture

hMPV NL/1/00 (virus genotype A) and NL/1/99 (virus genotype B) were kindly provided by Professor Fouchier (Erasmus Medical Center, Netherlands) and were prepared using the reverse genetics method as described previously^56,57^. Viruses were used to infect Vero-E6 cells, which were incubated at 37 °C and cultured in DMEM in the presence of 3% FBS (Invitrogen, USA), 2 mM L-glutamine, and 5 μg/ml trypsin (Sigma, USA). Five days after infection, the culture was ultra-centrifuged at 250,000 rpm at 4 °C for 12 h using a Beckman ultracentrifuge. NL/1/00 and NL/1/99 were respectively quantified by 50% tissue culture infectious doses (TCID50) and real-time PCR. The concentrations of NL/1/00 and NL/1/99 were 10^9^ copies/ml. Then, the two viral strains were mixed to generate three different proportions of hybrid virus strains at ratios of (A:B) 50:50, 20:80, 80:20, 10:100 and 100:10 on the basis of the real-time PCR results.

### *In vitro* competitive growth experiment

The *in vitro* competitive growth experiments were performed with conventional Vero E6 cells. Cells in 6-well plates were inoculated with viruses at the same MOI (MOI = 50). NL/1/00, NL/1/99 and mixed virus strains of five different proportions were inoculated into six wells along with a negative control (non-infection). Then, the plates were incubated at 37 °C for 1 h to allow the virus to attach. The cells were washed with phosphate-buffered saline (PBS) to remove any unattached infectious virus particles and then overlaid with 1 ml of maintenance medium. The plates were incubated at 37 °C in 5% CO_2_. When the cytopathogenic effect (CPE) was 70%, all supernatants and plates were harvested and stored at −80 °C. One millilitre of viral media was harvested at each passage, and the supernatant was passaged into fresh Vero-E6 cells. Then, 200 μl of RNA extract was added, and the remaining volume was maintained at −80 °C. This method was used for each passage and was continued for 12 passages. After each passage, we changed the medium and harvested the supernatant for testing. All tests were performed in triplicate, and six-well plates were used.

### *In vivo* competitive growth experiment

We carried out the test in batches because of the large number of animals. BALB/c mice (6–8 weeks old, female) were purchased from the Experimental Animal Center of Chongqing Medical University and were divided into six groups: 54 mice each experimental group(A, B, C, D, E) and 6 mice in control group(F). Each mouse was infected intranasally with 50 μl 1.0 × 10^9^ copies/ml of virus after being anesthetized with chloral hydrate. Group A received NL/1/00, group B received NL/1/99, group C received a 50:50 mixture of NL/1/00 and NL/1/99, group D received a 20:80 mixture of strains, group E received a 80:20 mixture of strains, and group F was the control group and received 50 μl DMEM. The mice were housed in groups in individual ventilated cages (IVC). Each group of mice were sacrificed at different times post infection (1, 2, 4, 5, 6, 8, 13, 17, and 20 days); 6 mice were sacrificed every time from each group, the lung tissue of the mice were extracted to measure viral load. This competition experiments found that the clinical manifestations of mice were most obvious on the fourth and fifth days after hMPV infection, so airway responsiveness experiments were performed on the fourth day after infection, and lung tissue was extracted on the fifth day for pulmonary histopathology.

### Noninvasive measurement of airway responsiveness

Approximately 36 BALB/c mice (6–8 weeks old, female) were divided into 6 groups: A, B, C, D, E, and F, 6 mice pre group. Four days after infection, each mouse was placed into a whole body plethysmography chamber (EMKA, France) and was nebulized first with PBS then with increasing doses (3.125, 6.25, 12.5, 25, and 50 mg/ml) of methacholine for 3 minutes for each nebulization; the mice were allowed to rest for 2 minutes, which was followed by the measurement of the breathing parameters for 5 minutes after each nebulization to determine the Pehn values.

### Pulmonary histopathology

Approximately 36 BALB/c mice (6–8 weeks old, female) were divided into 6 groups: A, B, C, D, E, and F, 6 mice pre group. Five days after infection, the left lungs of BALB/c mice were removed and fixed with 10% buffered formalin. The fixed lungs were embedded in paraffin, sectioned in 4 μm slices, and stained with haematoxylin and eosin. Four types of histopathological changes were scored for each section: peri bronchiolitis (inflammatory cells in the surrounding bronchiole), perivasculitis (inflammatory cells in the surrounding blood vessel), interstitial pneumonitis (increased thickness of the alveolar walls associated with inflammatory cells), and alveolitis (inflammatory cells within alveolar spaces). Each histopathological change was scored based on a numerical score ranging from 0–26^58^. The final score for each animal (ranging from 0–26) was obtained by averaging the scores for each lung, which were calculated by the addition of the subscores obtained from the assessment of the quantity and quality of the peribronchiolar and peribronchial infiltrates, luminal exudates, and perivascular infiltrates

### RNA extraction and cDNA synthesis

On days 1, 2, 4, 5, 6, 8, 13, 17, and 20 post infection, the animals were sacrificed, the lungs were removed, and 20 mg of tissue was weighed out and quickly homogenized. Viral RNA was extracted from 200 μl of lung homogenate using the QIAamp viral RNA mini kit (Qiagen, Germany) according to the manufacturer’s protocol, with elution in a final volume of 40 μl. According to the manufacturer’s instructions, complementary DNA (cDNA) was synthesized from 20 μl of eluted RNA using the PrimeScript RT Reagent Kit (TaKaRa, Dalian, China).

### Real-time PCR

For the detection of hMPV subtype A and B, a TaqMan probe-based real-time PCR method was used. All protocols utilized the methods of Professor Fouchier RA. The mixtures were processed prior to PCR amplification with the CFX96™ Real-Time PCR Detection System (Bio-Rad Laboratories). The amplification data were collected and analysed with CFX Manager^TM^ Software version 2.0 (Bio-Rad Laboratories, Hercules, USA).

### PCR and sequencing

We used direct and clone-sequencing methods to verify the real-time PCR results when detecting mixed infections of two hMPV genotypes. The amplified products were analysed by electrophoresis on a 2% agarose gel stained with ethidium bromide (EB), and the sizes of the amplified fragments were compared with those of standard molecular weight markers. To validate the amplification process and to exclude the presence of carryover contamination, positive and negative controls were included in each PCR. The amplified fragments were purified with a QIAquick PCR purification kit (Qiagen, Germany). The sequences were determined using an ABI Prism 3730 XL automated capillary DNA sequencer located at the company of Sangon (Shanghai, China).

### Animal weight

At different times post infection (0, 1, 2, 4, 6, 8, 13 and 20 days), the weight of every mouse in each of the groups was recorded.

### Ethics statement

The Chongqing Science and Technology Commission approved the production and the use of the Experimental Animal Center of Chongqing Medical University. The production license number is SCXK-(Yu)2018-0003, and the license number is SYXK(Yu)2018-0003. The animal care and use was performed according to the “Regulations on the Management of Laboratory Animals” of the Ministry of Science and Technology and the National Laboratory for Quality and Technical Supervision (GB14922-2001 to GBT14927-2001). The animal experiments were approved by the Experimental Animal Center of Chongqing Medical University. The experimental protocol was approved by the Children’s Hospital of Chongqing Medical University.

### Statistical analysis

Data obtained from the competitive replication experiments are expressed as the mean and standard deviation. Mixed linear models were used to compare the weight changes and viral loads after infection in different groups of animals at different time points. General linear models were used for comparisons of data obtained from noninvasive measurements of airway responsiveness. A rank correlation test was used for the analysis of the data for pulmonary histopathology.

## Data Availability

All data from this study are available.
